# c-FOS Confers Stem Cell-like Features to Multiple Myeloma Cells in a Bone Marrow Microenvironment

**DOI:** 10.3390/cells14070474

**Published:** 2025-03-21

**Authors:** Naoki Osada, Jiro Kikuchi, Sae Matsuoka, Hiroshi Yasui, Sho Ikeda, Naoto Takahashi, Yusuke Furukawa, Hideki Nakasone

**Affiliations:** 1 Division of Emerging Medicine for Integrated Therapeutics (EMIT), Center for Molecular Medicine, Jichi Medical University, Shimotsuke 329-0498, Japan; n_osada@jichi.ac.jp (N.O.); saematsuoka690@gmail.com (S.M.); furuyu@jichi.ac.jp (Y.F.); nakasone-tky@outlook.com (H.N.); 2 Department of Hematology & Oncology, St. Marianna University School of Medicine, Kawasaki 216-8511, Japan; hiroyasu@g.ecc.u-tokyo.ac.jp; 3 Department of Hematology/Oncology, The Institute of Medical Science, The University of Tokyo, Minato, Tokyo 108-8639, Japan; 4 Department of Hematology, Nephrology and Rheumatology, Akita University Graduate School of Medicine, Akita 010-8543, Japan; sikeda@med.akita-u.ac.jp (S.I.); naotot@doc.med.akita-u.ac.jp (N.T.); 5 Center for Medical Education, Teikyo University of Science, Tokyo 120-0045, Japan

**Keywords:** c-FOS, drug resistance, cancer stem cell, bone marrow microenvironment, multiple myeloma

## Abstract

Multiple myeloma (MM) is the second most common hematologic malignancy and has a poor prognosis. Although the outcomes of MM have markedly improved with the approval of novel agents, the high incidence of relapse means that MM remains incurable. The bone marrow microenvironment (BMME) contributes to drug resistance and minimal residual disease (MRD), which is a major source of relapse in patients with MM. However, the underlying molecular mechanisms are not fully understood. We have previously shown that the upregulation of the AP-1 transcription factor c-FOS confers lenalidomide resistance by maintaining IRF4 expression in MM cells. In this study, we show that upregulated expression of c-FOS confers a poor prognosis and cancer stem cell-like features, including drug resistance, within BMME, both in vitro and in vivo, via IRF4 upregulation; and that inhibition of c-FOS by the AP-1 inhibitor, T-5224, prevents regeneration of MM cells via IRF4 downregulation in a murine serial transplantation assay. These results suggest a functional role for c-FOS in conferring cancer stem cell-like features to MM cells in the BMME for the first time. Therefore, c-FOS inhibition may be an effective treatment strategy for improving the outcomes of patients with MM by eliminating drug-resistant cancer stem cell-like MM cells in MRD.

## 1. Introduction

Multiple myeloma (MM) is the second most common hematologic malignancy and has a poor prognosis. Recently, the outcomes of MM have markedly improved with the approval of novel agents such as proteasome inhibitors, immunomodulatory drugs, and monoclonal antibodies. However, the high incidence of relapse means that MM remains incurable [1]. It is widely accepted that relapse is initiated by drug-resistant residual MM cells (minimal residual disease; MRD) in the bone marrow (BM) niche [2]. Recent studies have revealed that MRD significantly increases the risk of relapse and reduces overall survival (OS) rates. Therefore, achieving and maintaining MRD negativity has become a critical goal in the treatment of MM [3].

The interaction between MM cells and the BM microenvironment (BMME) confers drug resistance [4], adaptation to hypoxic environments [5], dormancy [5,6], and the ability to regenerate within the BMME [7], which are major characteristics similar to those of cancer stem cells [8]. Therefore, MM stem-like cells (MMSCs) can be generated and maintained within the BMME and are a source of MRD and clonal evolution. Thus, the elimination of MMSCs may be an effective way to achieve MRD negativity.

The activator protein-1 (AP-1) family transcription factors (TFs), composed of JUN and FOS proteins, play essential roles in both physiological processes and tumorigenesis. Of these, we have shown that c-FOS, a member of the AP-1 family, and its heterodimeric partner JUN, but not FOSB, are expressed at a higher level in MM cells compared to normal plasma cells; c-FOS is an integral component of the IKZF1 complex and is primarily responsible for the activator function of the complex in MM cells; c-FOS binds to DNA through protein-protein interactions with IKZF1 and forms MM-specific IKZF complex, composed of IKZF1/IKZF3 and c-FOS/JUN, to enhance the transcription of IKZF target genes including IRF4 and SLAMF7; the upregulation of c-FOS confers lenalidomide resistance by maintaining IRF4 expression regardless of IKZF1 downregulation [9].

A recent study revealed that the interaction between MM cells and BM stromal cells (BMSCs)-induced gene expression showed independent prognostic significance in MM. Potential target genes were predicted to bind a number of TFs, including FOS and IRF family members [10]. Using a patient-derived xenograft (PDX) murine model, another study showed that IRF4 supports the regenerative ability of MMSCs in the BMME [7]. In addition, previous studies have revealed that upregulated c-FOS expression induces dormancy in hematopoietic stem cells (HSCs) [11,12]. These findings prompted us to speculate the role of c-FOS in generating and maintaining MMSCs within the BMME. However, this role remains unclear.

In this study, we found that upregulated expression of c-FOS confers a poor prognosis and stem cell-like features, including drug resistance, to MM cells within the BMME, both in vitro and in vivo, via IRF4 upregulation. In addition, we showed that inhibition of c-FOS by the AP-1 inhibitor T-5224 prevents regeneration of MM cells in the BMME via IRF4 downregulation in a murine xenograft model. These results suggest that c-FOS inhibition may be a novel and effective treatment strategy for improving the outcomes of patients with MM by eliminating drug-resistant cancer stem cell-like MM cells in MRD.

## 2. Materials and Methods

### 2.1. Drugs

We purchased T-5224 from Selleck Chemicals (Houston, TX, USA). Drugs were dissolved in dimethyl sulfoxide (DMSO) and used at a final dilution of 1/1000 to maintain a final concentration of DMSO < 0.1% to prevent alterations in drug effects or cell growth.

### 2.2. Cells and Cell Culture

We used human MM cell lines, KMS12-BM, KMS21, and MM.1S, and sublines adapted to hypoxic conditions, KMS12-BM-Low, KMS21-Low, and MM.1S-low, in this study. These were purchased from the Health Science Research Resources Bank (Osaka, Japan), where cell line authenticity and Mycoplasma infection were routinely checked by DNA fingerprinting and PCR. We used the human BM-derived stromal cell line UBE6T-7, which was immortalized by transduction with a telomerase catalytic protein subunit, as BMSCs [4].

### 2.3. In Vitro Co-Culture System with BMSCs to Mimic the BM Microenvironment

First, BMSCs were cultured on the reverse side of the polyethylene terephthalate track-etched membrane of a high-pore-density cell culture insert (35–3495; Becton-Dickinson, Franklin Lakes, NJ, USA) in a 24-well plate (35–3504; Becton-Dickinson). After obtaining a confluent feeder layer, MM cells were seeded on the upper side of the membrane, where the cytoplasmic villi of BMSCs passed through the etched 0.4-mm pores. In another set of conditions, MM cells were seeded on the upper side of a low-pore-density cell culture insert (35–3095; Becton-Dickinson). Under these conditions, MM cells are physically separated from the stromal layer, providing an adhesion-negative control [4]. We performed co-culture under hypoxic (5% O_2_) conditions to mimic BMME.

### 2.4. Quantitative Real-Time Reverse Transcription-PCR (qPCR)

Total cellular RNA was isolated using an RNeasy Kit (Qiagen, Venlo, The Netherlands), reverse-transcribed into complementary DNA using ReverTra Ace and oligo (dT) primers (Toyobo, Osaka, Japan), and subjected to quantitative real-time reverse transcription-PCR using Expression Assays (Hs00958474 for IKZF1, Hs99999140 for c-FOS, Hs00180031 for IRF4, Hs00904275 for SLAMF7, and Hs01922876 for GAPDH) and TaqMan Fast Universal PCR Master Mix [9].

### 2.5. Immunoblotting

Immunoblotting was performed as standard methods using antibodies against IKZF1 (#14859), c-FOS (#2250), IRF4 (#15106), SLAMF7 (#98611) (Cell Signaling Technology, Beverly, MA, USA), Oct4 (Invitrogen, Waltham, MA, USA), SOX2 (NOVUS, Littleton, CO, USA), NANOG (ReproCELL, Tokyo, Japan), and GAPDH (sc-47724) (Santa Cruz Biotechnology, Dallas, TX, USA) [13].

### 2.6. Cell Proliferation Assay

Cell proliferation was monitored using a Cell Counting Kit (Wako Chemicals, Osaka, Japan), in which the absorbance of reduced 3-(4,5-dimethylthiazol-2-yl)-5-(3-carboxymethophenyl-2-(4-sulfophenyl)-2H-tetrazolium (MTT) is proportional to viable cell numbers [13].

### 2.7. Chromatin Immunoprecipitation (ChIP) Assay

We used the ChIP-IT Express Enzymatic Kit (Active Motif, Carlsbad, CA, USA) to perform ChIP assays. In brief, we fixed cells in 1% formaldehyde at room temperature for 10 min and isolated chromatin fractions using sonicated chromatin suspensions. After centrifugation, the supernatants were incubated with antibodies or isotype-matched controls in the presence of protein G magnetic beads at 4 °C overnight. We purified DNA fragments from the mixture and carried out quantitative PCR using Power SYBR Green Master Mix (Thermo Fisher Scientific, Waltham, MA, USA) and the primers. The data were normalized to the values of the input, quantified using the 2^−∆∆Ct^ method, and are shown as fold increases against the values obtained with control IgG immunoprecipitants [9].

### 2.8. Construction and Production of Lentiviral Expression Vector

We used a lentiviral vector, CSII-CMV-MCS-IRES-VENUS (provided by Dr. Hiroyuki Miyoshi, RIKEN BioResource Center, Ibaraki, Japan), which contained the coding region of *IRF4* cDNA. The vector was co-transfected into 293FT cells with packaging plasmids (Invitrogen) to produce infective lentiviruses in the culture supernatant [13].

### 2.9. Murine Xenograft MM Model

We transplanted MM.1S-Luc cells (1 × 10^6^ cells/mouse) into NOD/SCID mice (CLEA Japan, Shizuoka, Japan) via the tail vein. After confirming the engraftment of transplanted cells in the BM, usually on day 7, the mice were assigned to two groups (n = 5) by measuring tumor-derived luciferase activity, standardized on the basis of total luciferase activity, using the IVIS Imaging System with Living Image software version 4.8.2 (Xenogen, Alameda, CA, USA): treatment with vehicle alone (DMSO, three times a week) or 20 mg/kg T-5224 (three times a week) intraperitoneally for 3 weeks. After 4 weeks, the femurs and tibiae of the mice were dissected and cleaned of the surrounding tissue. The distal and proximal ends were opened, and the BM was flushed with ice-cold phosphate-buffered saline. Finally, MM cells were isolated from BM cells using anti-human CD138 MicroBeads and MACS separation columns (Miltenyi Biotech, Gladbach, Germany). Then, we serially transplanted the isolated cells (1 × 10^6^ cells/mouse) into other NOD/SCID mice via the tail vein [8]. All animal studies were approved by the Institutional Animal Ethics Committee and performed in accordance with the Guide for the Care and Use of Laboratory Animals, formulated by the National Academy of Sciences.

### 2.10. Statistics

We used KaleidaGraph software version 4.5.3 (Synergy Software, Reading, PA, USA) to perform one-way analysis of variance (ANOVA) with the Student−Newman−Keuls multiple comparison test and Student’s *t*-test to determine statistical significance. *p*-values less than 0.05 were considered statistically significant.

## 3. Results

### 3.1. Upregulation of c-FOS Expression Confers Poor Prognosis in Patients with MM

**Figure 1:**
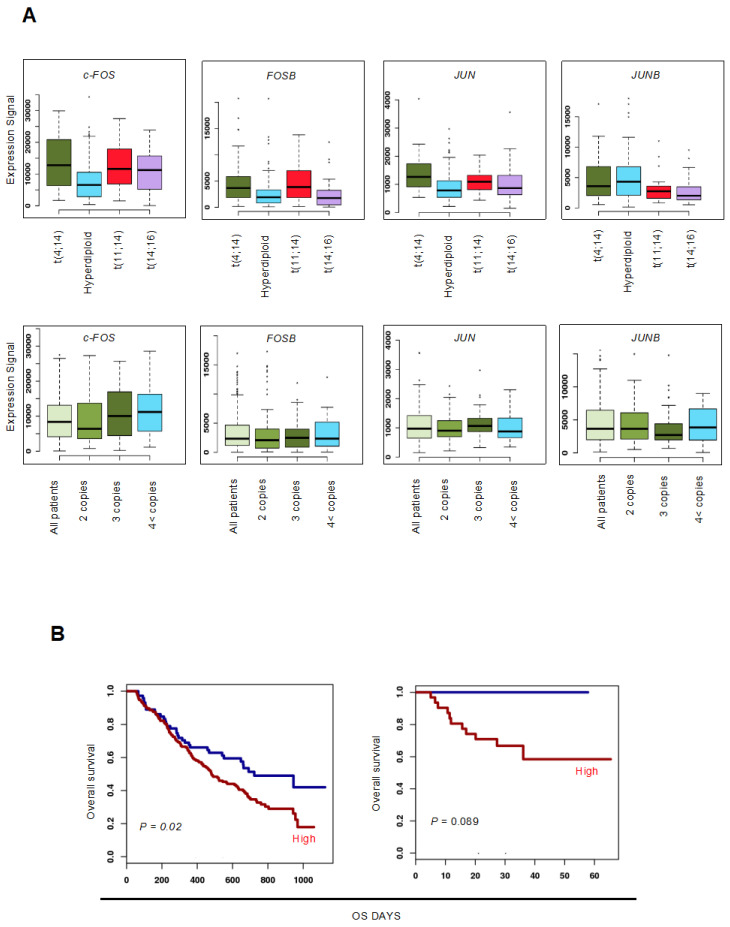
Upregulation of c-FOS expression is associated with poor prognosis in patients with MM. (**A**) Using the GenomicScape tool (www.genomicscape.com), we examined the expression of AP-1 family genes (*c-FOS*, *FOSB*, *JUN*, and *JUNB*) in MM cells with t(4;14), hyperdiploid, t(11;14), and t(14;16) (upper panels) and a copy number of 1q21 (lower panels) [GSE4581]. (**B**) Kaplan–Meier curves of patients with MM showing high (n = 192; red) and low (n = 72; blue) expression of *c-FOS* with bortezomib monotherapy (left panel). Kaplan–Meier curves of patients with MM harboring 1q amp (4 < copies) showing high (n = 31; red) and low (n = 7; blue) expression of c-FOS with total therapy 2 and 3 (right panel). *p*-values were determined using the log-rank test.

First, we determined the expression levels of AP-1 family TFs and their prognostic effects in patients with MM. When compared to MM cells with hyperdiploid or without amplification of chromosome 1q21 (1q gain/amp), we found that c-FOS expression was significantly higher in MM cells carrying high-risk chromosomal abnormalities, including t(4;14), t(14;16), and 1q gain/amp. In contrast, the expression levels of FOSB and JUN were higher in MM cells carrying t(4;14) but not t(14;16) and 1q gain/amp. However, we did not observe upregulation of JUNB in MM cells with high-risk chromosomal abnormalities (Figure 1A). The OS of patients with MM showing higher expression of c-FOS was significantly shorter than that of those showing lower expression when treated with bortezomib monotherapy or total therapy 2 and 3 (TT 2/3), which includes bortezomib, dexamethasone, thalidomide, and other chemotherapeutic agents (Figure 1B) [14]. In addition, we have shown that c-FOS is expressed at a higher level in MM cells and most MM cell lines compared to normal plasma cells [9]. These results suggest that the upregulation of c-FOS mediates poor prognosis in patients with MM. Therefore, we focused on c-FOS in a further study.

### 3.2. Upregulation of c-FOS Expression in MM Cells Under Co-Cultured with BMSCs and in the BMME of a Xenograft Murine Model

**Figure 2:**
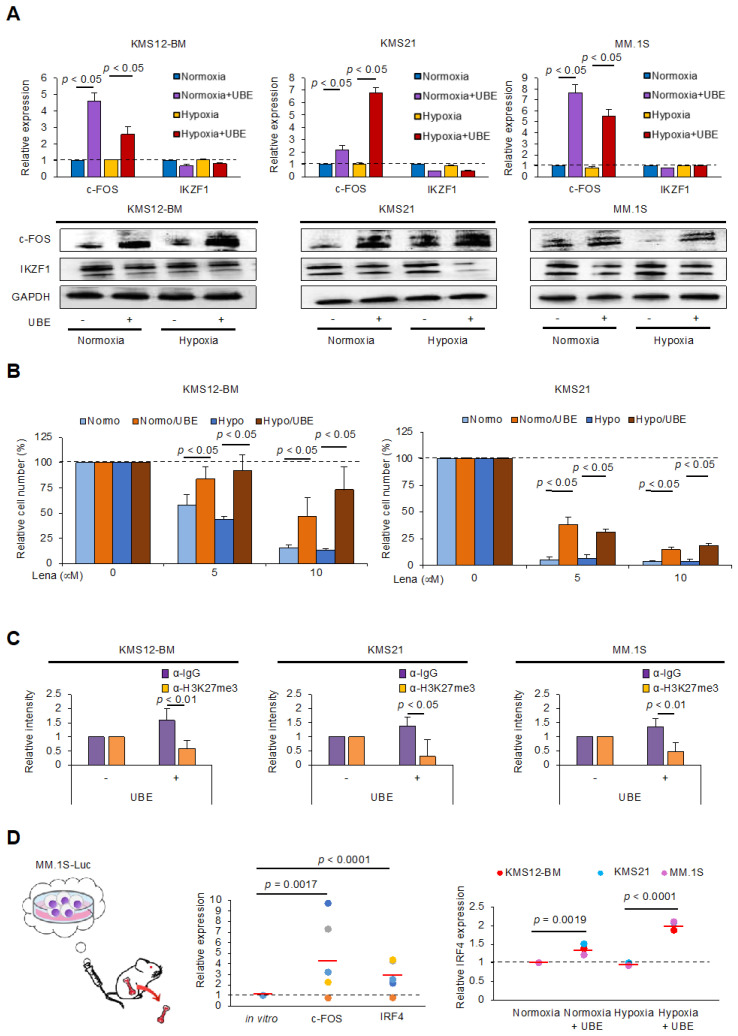
Upregulation of c-FOS expression in MM cells co-cultured with BMSCs and in the BMME of a xenograft murine model. (**A**) MM cell lines were cultured with or without adhesion to UBE6T-7 cells (UBE) under normoxic (20% O_2_) (Normoxia) or hypoxic (5% O_2_) (Hypoxia) conditions for 72 h. The expression levels of *IKZF1* and *c-FOS* mRNAs were determined by qPCR relative to normoxia values set at 1.0. Data were quantified using the 2^−∆∆Ct^ method, with *GAPDH* as a reference. The means ± S.D. (bars) of three independent experiments are shown. *p*-values were determined using one-way ANOVA with the Student−Newman−Keuls multiple comparison test (upper panels). Whole cell lysates were simultaneously prepared and subjected to immunoblotting for IKZF1, c-FOS, and GAPDH proteins (lower panels). (**B**) MM cells were cultured with or without adhesion to UBE6T-7 cells (UBE) under normoxic (20% O_2_) (Normo) or hypoxic (5% O_2_) (Hypo) conditions and treated with various concentrations of lenalidomide (Lena) for 72 h. Cell proliferation was estimated using the MTT assay and was expressed as a percentage of the absorbance value of the corresponding untreated cells. The left and right panels show the results obtained from KMS12-BM and KMS21 cells, respectively. The means ± S.D. (bars) of three independent experiments are shown. *p*-values were determined using one-way ANOVA with the Student−Newman−Keuls multiple comparison test. (**C**) Chromatin suspensions were prepared from MM cells cultured with or without adhesion to UBE6T-7 cells (UBE) under normoxic condition for 72 h and immunoprecipitated with specific antibody against H3K27me3 (α-H3K27me3) or isotype-matched IgG (α-IgG). The resulting precipitates were subjected to qPCR to amplify the regions of the *c-FOS* promoter (forward: 5′-CCCAGCAGTCGAGGTATTCC-3′ and reverse: 5′-CCTGCGGGGAACTCAAATCT-3′). The data were normalized to the values of input, quantified by the 2^−∆∆Ct^ method, and are shown as fold increases against the values obtained with IgG immunoprecipitants from cell samples without adhesion (UBE [-]). The means ± S.D. (bars) of three independent experiments are shown. *p*-values were determined using one-way ANOVA with the Student−Newman−Keuls multiple comparison test. (**D**) We transplanted luciferase-expressing MM.1S cells (MM.1S-Luc; 1 × 10^6^ cells/mouse) into the NOD/SCID mice via tail veins. After 4 weeks, the femurs and tibiae of mice were dissected and cleaned of surrounding tissue. The distal and proximal ends were opened, and the BM was flushed using ice-cold phosphate-buffered saline. Finally, MM cells were isolated from BM cells using anti-human CD138 MicroBeads and MACS separation columns (left panel). Then, we determined the mRNA expression levels of *c-FOS* and *IRF4* by qPCR relative to the values before transplantation (in vitro) set at 1.0. Data were quantified using the 2^−∆∆Ct^ method with *GAPDH* as a reference. Bars indicate the mean values of five mice, each shown in a different color (middle panel). MM cell lines were cultured with or without adhesion to UBE6T-7 cells (UBE) under normoxic (20% O_2_) (Normoxia) or hypoxic (5% O_2_) (Hypoxia) conditions for 24 h. The expression level of *IRF4* mRNA was determined by qPCR relative to normoxia values, set at 1.0. Data were quantified using the 2^−∆∆Ct^ method with *GAPDH* as a reference. Bars indicate the mean values of the three MM cell lines (right panel). *p*-values were determined by one-way ANOVA with the Student–Newman–Keuls multiple comparison test.

Next, we determined the expression of c-FOS in MM cells in the BMME. Previous studies have revealed that dormant MM cells physically interact with BMSCs under hypoxic conditions within the BMME [6,15]. Therefore, we conducted experiments under both normoxic and hypoxic conditions. We co-cultured MM cell lines and sublines adapted to hypoxic conditions with direct adhesion to the BM stromal cell line, UBE6T-7, under normoxic (20% O_2_) or hypoxic (5% O_2_) conditions to mimic the BMME in vitro. Subsequent quantitative real-time reverse transcription-PCR (qPCR) and immunoblot analyses revealed the upregulation of c-FOS expression, but not IKZF1, with adhesion under either normoxic or hypoxic conditions, whereas no significant differences were observed between normoxic and hypoxic conditions (Figure 2A). Concurrent with c-FOS upregulation, adhesion to BMSCs induced significant lenalidomide resistance in MM cells (Figure 2B). We have previously shown that adhesion to BMSCs epigenetically upregulates the expression of anti-apoptotic genes and induces drug resistance in MM cells via downregulation of histone H3 lysine 27 trimethylation (H3K27me3), a critical repressive histone mark [4]. We subsequently performed a chromatin immunoprecipitation assay using an anti-H3K27me3 antibody and revealed significant downregulation of H3K27me3 in the promoter region of the c-FOS gene (Figure 2C). These results suggest that adhesion to BMSCs upregulates c-FOS expression in MM cells via hypomethylation of H3K27me3 in the promoter region.

Based on these findings, we determined c-FOS expression in MM cells in vivo. In this experiment, we established a murine xenograft model using MM.1S-Luc cells injected via the tail vein. After transplantation, MM.1S-Luc cells became widely distributed within the sacrum, vertebrae, ribs, and femurs, which faithfully reproduces human MM. Subsequently, we obtained MM cells from the femurs of recipient mice and determined the expression of c-FOS. As anticipated, qPCR analyses revealed the upregulation of c-FOS in MM cells derived from the femur compared to that before transplantation (Figure 2D). However, previous studies have shown that IRF4 is a key molecule in MMSC regeneration [7] and a downstream target of c-FOS in MM cells [9]. We therefore determined IRF4 expression and revealed its significant upregulation both in vitro and in vivo (Figure 2D). Previous studies have revealed that hypoxic BMME confers stem cell-like features to MM cells, coinciding with the upregulation of stem cell markers such as Oct4, SOX2, and NANOG [16,17]. Among them, we found c-FOS binding to the promoter/enhancer regions of Oct4 and SOX2 genes in the ChIP-sequence data [GSE194381] (Supplemental Figure S1A) [9]. The OS of patients with MM showing higher expression of Oct4 or SOX2 was shorter than that of those showing lower expression, when treated with TT 2/3 (Supplemental Figure S1B). Furthermore, we found the upregulation of Oct4 and SOX2 expression with adhesion to BMSCs under hypoxic conditions in KMS12-BM and KMS21 cells (Supplemental Figure S1C). Taken together, these results suggest that adhesion to BMSCs increases the expression of c-FOS and its downstream targets, including IRF4, Oct4, and SOX2, which may confer drug resistance and stem cell-like features to MM cells in the BMME.

### 3.3. AP-1 Inhibitor Prevents Regeneration of MM Cells in BMME via IRF4 Downregulation

**Figure 3:**
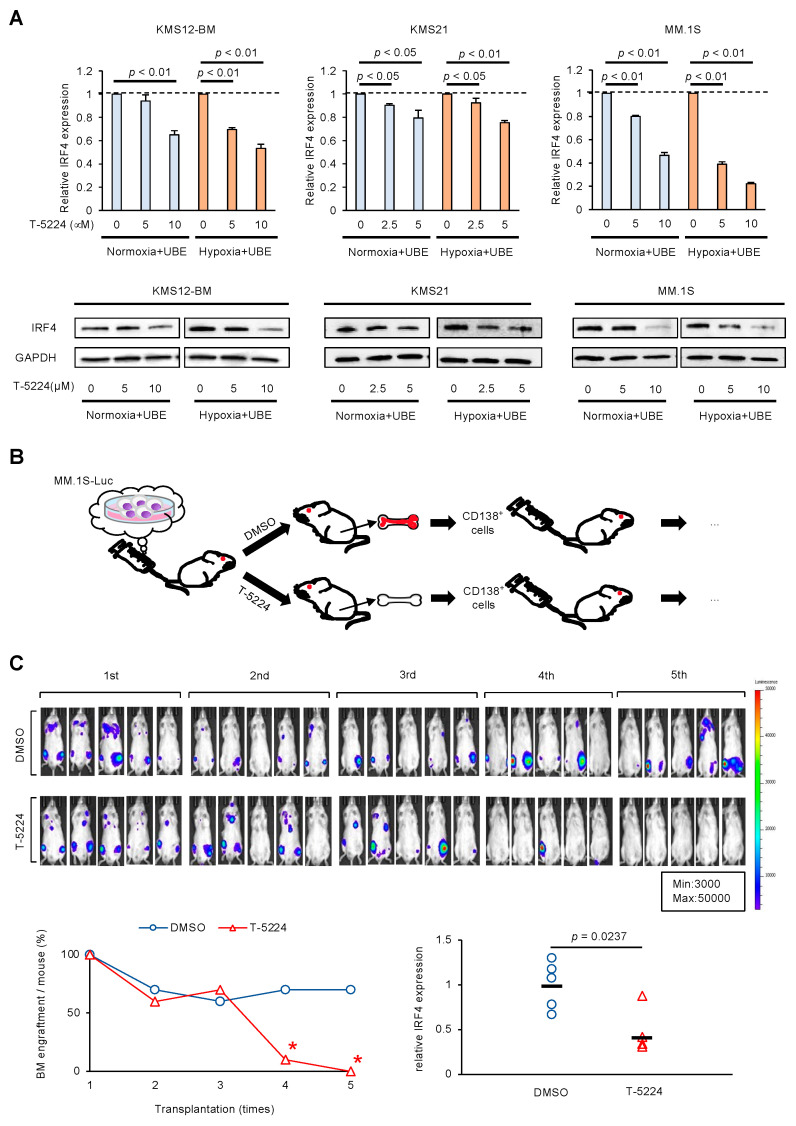
AP-1 inhibitor prevents regeneration of MM cells in the BMME via IRF4 downregulation. (**A**) MM cell lines were co-cultured with adhesion to UBE6T-7 cells under normoxic (Normoxia + UBE) or hypoxic (Hypoxia + UBE) conditions and treated with various concentrations of T-5224 for 72 h. The mRNA expression level of *IRF4* was determined by qPCR and is shown relative to the untreated control values set at 1.0. Data were quantified using the 2^−∆∆Ct^ method with *GAPDH* as a reference. The means ± S.D. (bars) of three independent experiments. *p*-values were determined using one-way ANOVA with the Student−Newman−Keuls multiple comparison test (upper panels). Whole cell lysates were simultaneously prepared and subjected to immunoblotting for IRF4 and GAPDH proteins (lower panels). (**B**) We transplanted MM.1S-Luc cells (1 × 10^6^ cells/mouse) into NOD/SCID mice via the tail vein. After confirming the engraftment of transplanted cells in the BM, usually on day 7, the mice were assigned to two groups by measuring tumor-derived luciferase activity, standardized on the basis of total luciferase activity, with a noninvasive bioimaging system: treatment with vehicle alone (DMSO, three times a week) (n = 5) or 20 mg/kg T-5224 (three times a week) (n = 5) intraperitoneally for 3 weeks. After 4 weeks, the femurs and tibiae of mice were dissected and cleaned of surrounding tissue. The distal and proximal ends were opened, and the BM was flushed using ice-cold phosphate-buffered saline. Finally, MM cells were isolated from BM cells using anti-human CD138 MicroBeads and MACS separation columns. Then, we serially transplanted the isolated cells (1 × 10^6^ cells/mouse) into other NOD/SCID mice via the tail vein. (**C**) Ex vivo bioluminescence imaging was performed 4 weeks after each transplantation. The numbers 1st, 2nd, 3rd, 4th, and 5th refer to the serial transplantations (upper panels). The average engraftment rates are shown for the indicated periods. The engraftment rate was as follows: engraftment in both left and right femurs and tibia, 100%; engraftment in either femur or tibia, 50%; and no engraftment, 0%. * *p* < 0.05 by one-way ANOVA with a Student–Newman–Keuls multiple comparison test (lower left panel). Total RNA was isolated from MM cells engrafted in the femurs and tibiae of recipient mice after treatment with vehicle control (DMSO) or 20 mg/kg T-5224 three times a week, intraperitoneally, for 3 weeks. The expression level of *IRF4* was measured by qPCR and is shown relative to the average value of vehicle control (DMSO), set as 1.0. Data were quantified using the 2^−∆∆Ct^ method with *GAPDH* as a reference. Bars indicate the mean values of five mice (lower right panel). *p*-values were determined using the Student’s *t*-test.

Our present findings prompted us to speculate that the inhibition of c-FOS could prevent the regeneration of MM cells in the BMME via downregulation of IRF4. We then determined the effect of the AP-1 inhibitor T-5224 on IRF4 expression in MM cells in vitro. As anticipated, qPCR and immunoblot analyses revealed that T-5224 significantly downregulated IRF4 expression in MM cells in a dose-dependent manner with adhesion to BMSCs under both normoxic and hypoxic conditions (Figure 3A). Coinciding with IRF4 downregulation, T-5224 conferred cytotoxicity in a dose-dependent manner, whereas forced expression of IRF4 mitigated the effect in KMS12-BM and MM.1S cells (Supplemental Figure S2). These results suggest that IRF4 is a critical target of T-5224 in MM cells. Next, we determined the effect of T-5224 on the regenerative ability of MM cells in vivo. To this end, we performed a serial transplantation assay, which is a method to assess the pluripotency and regenerative ability of HSCs and cancer stem cells [8]. We obtained MM cells from the femurs of recipient mice after treatment with dimethyl sulfoxide (Control) or T-5224 and serially transplanted these into other mice via tail veins (Figure 3B). In our previous study, we failed to prolong the survival of KMS-26-transplanted mice treated with T-5224 twice a week, probably due to IRF4 upregulation in BMME [9]. Therefore, in this study, we increased the frequency of administration to three times a week for three weeks. As a result, MM cells successfully regenerated in the BM, even after serial transplantation, in control mice. This suggests that the BMME confers stemness to MM cells via c-FOS upregulation. However, T-5224 significantly reduced the regenerative ability of MM cells after the fourth transplantation, coinciding with the significant downregulation of IRF4 expression in MM cells (Figure 3C). These results suggest that inhibition of c-FOS may prevent the generation of MM cells with stem cell-like features in the BMME via downregulation of IRF4 expression.

### 3.4. AP-1 Inhibitor Reduces the Expression of SLAMF7 in MM Cells Under Adhesion to BMSCs

**Figure 4:**
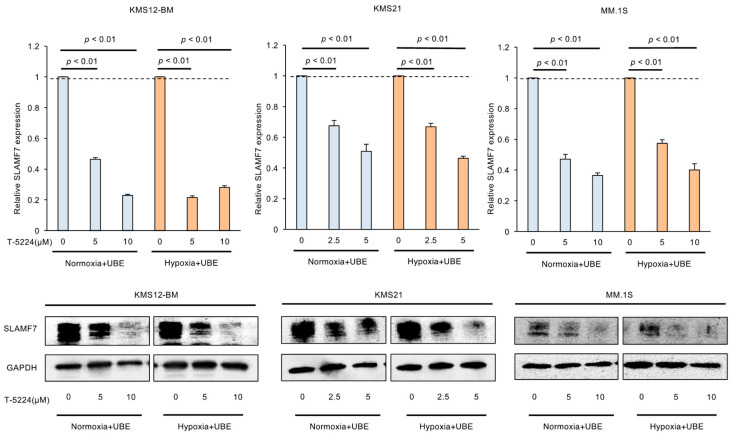
MM cell lines were co-cultured with adhesion to UBE6T-7 cells under normoxic (20% O_2_) (Normoxia + UBE) or hypoxic (5% O_2_) (Hypoxia + UBE) conditions and treated with various concentrations of T-5224 for 72 h. The mRNA expression level of *SLAMF7* was determined by qPCR and is shown relative to the untreated control values set at 1.0. Data were quantified using the 2^−∆∆Ct^ method with *GAPDH* as a reference. The means ± S.D. (bars) of three independent experiments. *p*-values were determined by one-way ANOVA with the Student−Newman−Keuls multiple comparison test (**upper panels**). Whole cell lysates were simultaneously prepared and subjected to immunoblotting for SLAMF7 and GAPDH proteins (**lower panels**).

We have also shown that SLAMF7 is another downstream target of c-FOS in MM cells [9]. As anticipated, T-5224 reduced the expression of SLAMF7 in MM cells adhering to BMSCs (Figure 4). Since SLAMF7 mediates MM cell growth and immune suppression via the expansion of tumor-associated macrophages and regulatory T cells [18,19,20,21], inhibition of c-FOS may improve the immune microenvironment.

## 4. Discussion

We have previously shown that c-FOS is an integral component of the IKZF1 complex and is primarily responsible for its activator function in MM cells. Furthermore, the upregulation of c-FOS confers lenalidomide resistance by maintaining IRF4 expression. In contrast, T-5224, a selective AP-1 inhibitor, mitigates the activity of the MM-specific activator complex, resulting in the augmentation of the anti-MM effects of lenalidomide via IRF4 downregulation [9]. In the present study, we show that: (1) c-FOS upregulation confers a poor prognosis in patients with MM; (2) the expression of c-FOS and IRF4 is upregulated in MM cells within a hypoxic BMME, both in vitro and in vivo; and (3) inhibition of c-FOS by the AP-1 inhibitor T-5224 prevents the generation of MM cells with stem cell-like features in the BMME via IRF4 downregulation using a unique cell culture system to mimic the BMME and a serial transplantation assay. These results suggest that the BMME confers cancer stem cell-like features, including drug resistance to MM cells via the upregulation of c-FOS and IRF4 expression. In addition, the inhibition of c-FOS prevents the regeneration of MM cells in the BMME. This is the first report to demonstrate a functional role for c-FOS in conferring cancer stem cell-like features to MM cells within the BMME. Therefore, inhibition of c-FOS may be an effective treatment strategy to eliminate drug-resistant cancer stem cell-like MM cells in the BMME and achieve MRD negativity in patients with MM.

However, this study had several limitations. Our study lacked sufficient data to demonstrate the stem cell-like features. Therefore, additional experiments are needed to address this gap. For example, we performed most experiments using MM cell lines that represent advanced disease stages. The PDX murine model and analysis of the side population fraction may strengthen the claims of this study. We will begin these analyses in the near future.

A recent study revealed that the upregulation of c-FOS expression coincided with the activation of an integrin-mediated signaling pathway in residual MM cells after treatment with lenalidomide and dexamethasone [22]. In contrast, we have shown that adhesion to BMSCs significantly confers lenalidomide resistance to MM cells in vitro (Figure 2B), and that α4/β1 integrin is a key adhesion molecule in this drug resistance [4]. Therefore, in this case, we strongly postulate that residual MM cells include cancer stem cell-like MM cells within the BMME and may be the source of relapse.

Mondala et al. reported that inhibition of IRF4 by antisense oligonucleotide (ASO) prevented the regeneration of MMSCs in a PDX model [7]. Several clinical trials are currently in progress to determine the efficacy of ASOs and RNA interference in various diseases. However, several issues remain to be overcome, such as the development of delivery systems, management of off-target effects, and minimization of immune responses [23]. In contrast, we have shown that combination treatment with T-5224 and lenalidomide could significantly prolong the survival of recipient mice, in which MM cells were engrafted in the BM, without obvious toxicity [9]. Since both drugs can downregulate IRF4 expression in vitro [9], combination treatment might additively eliminate MMSCs in the BMME via downregulation of IRF4, resulting in prolonged survival of recipient mice. These results provide a rationale for the design of safe AP-1 inhibitor-based regimens that achieve MRD negativity by eliminating drug-resistant cancer stem cell-like MM cells and improving the outcome of MM.

## 5. Conclusions

Our present findings suggest a functional role of c-FOS in conferring cancer stem cell-like features to MM cells in the BMME for the first time and that c-FOS inhibition may be a novel and effective treatment strategy to improve the outcomes of MM patients by eliminating drug-resistant cancer stem cell-like MM cells in MRD. These results provide a rationale for the design of AP-1 inhibitor-based regimens that achieve MRD negativity and improve the outcome of MM.

## Data Availability

The data that support the findings of this study are available from the corresponding author upon reasonable request.

## Supplementary Materials

The following supporting information can be downloaded at: https://www.mdpi.com/article/10.3390/cells14070474/s1, Figure S1: Up-regulation of Oct4 and SOX2 expression is associated with poor prognosis in patients with MM and observed in MM cells with adhesion to BMSCs under hypoxic conditions; Figure S2: The forced expression of IRF4 mitigated the T-5224-induced cytotoxicity in MM cells.

## Abbreviations

The following abbreviations are used in this manuscript:

MM	multiple myeloma
MRD	Minimal residual disease
BMME	Bone marrow microenvironment
AP-1	Activator protein-1
