# Supplementary material for: c-FOS Confers Stem Cell-like Features to Multiple Myeloma Cells in a Bone Marrow Microenvironment
Source: Cells. 2025 Mar 21;14(7):474. doi: 10.3390/cells14070474 (PMC11987719; doi:10.3390/cells14070474)
Supplement: Supplementary file 1 [file cells-14-00474-s001.zip › cells-3480354-supplementary.pdf]

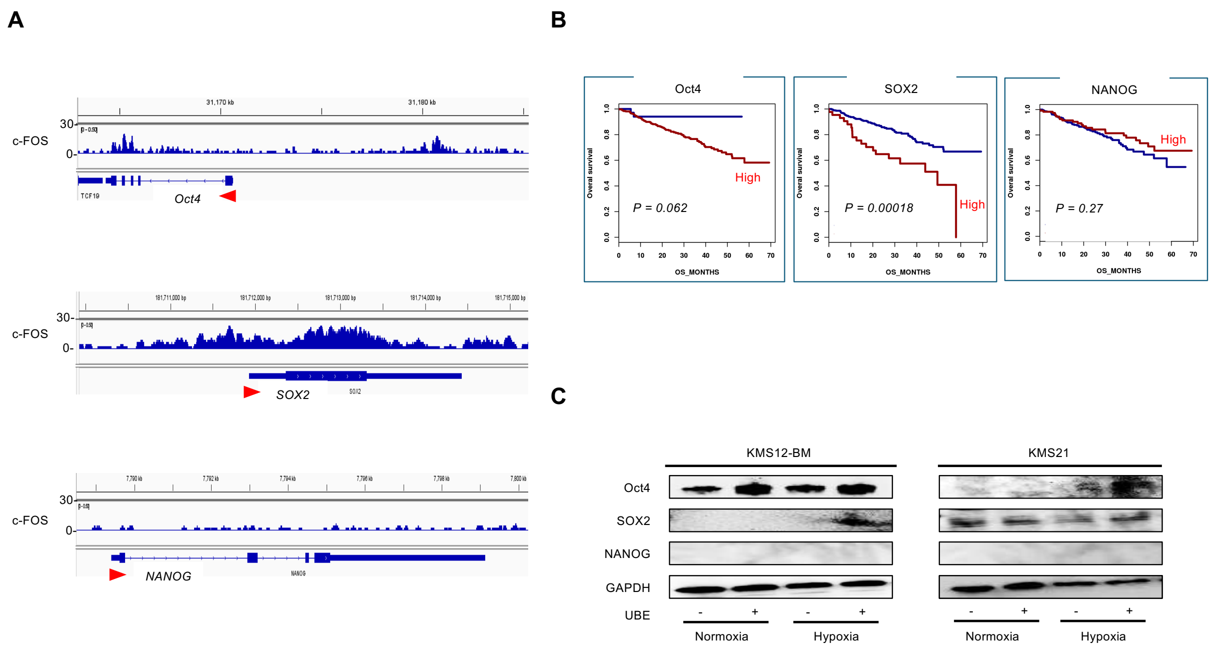

Supplemental Figure S1. (A) Using chromatin immunoprecipitation (ChIP)-seq data of MM.1S cells [GSE194381], we visualized c-FOS binding to the promoter/enhance regions of the indicated genes in the ChIP-Atlas browser. Red triangles indicate transcription start sites. (B) Kaplan–Meier curves of patients with MM showing high (red) and low (blue) expression of indicated genes, when treated with TT 2/3. *P* values were determined by a log-rank test. (C) KMS12-BM and KMS21 cells were cultured with or without adhesion to UBE6T-7 cells (UBE) under normoxic (20% O_2) (Normoxia) or hypoxic (5% O_2) (Hypoxia) conditions for 72 h. Whole cell lysates were prepared and subjected to immunoblotting for Oct4, SOX2, NANOG, and GAPDH proteins.

Supplemental Figure S2

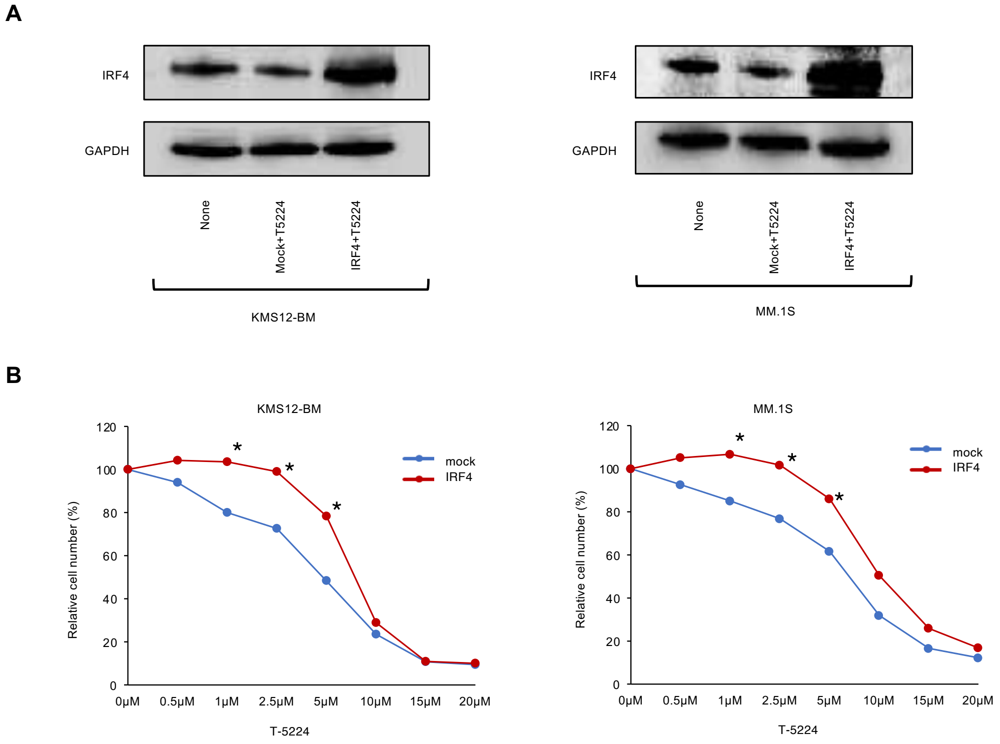

Supplemental Figure S2. (A) KMS12-BM and MM.1S cells were transduced with either an empty lentiviral vector (Mock) or IRF4-overexpressing vector (IRF4) to establish stable transformants. We cultured each transformant in the absence or presence of 5 μM T-5224 for 24 h, respectively. The expression level of IRF4 protein was determined by immunoblotting. (B) We cultured each transformant in the presence of T-5224 at the indicated concentrations for 72 h. Cell viability was determined by MTT reduction assay and expressed as a percentage of the values of untreated cells (n = 3). S.D. was less than 10% and thus omitted. *$P < 0.05$ by one-way ANOVA with a Student–Newman–Keuls multiple comparison test.
